# Comparative efficacy of unilateral biportal endoscopy versus endoscopic foraminoplasty decompression for single-level lumbar spinal stenosis: a retrospective cohort study

**DOI:** 10.3389/fsurg.2026.1857061

**Published:** 2026-07-29

**Authors:** Xu Qiao, Cao Rui, Aikebaierjiang Aisaiti, Li Ning, Shi Lingyun, Sheng Weibin

**Affiliations:** Spinal Surgery, The First Affiliated Hospital of Xinjiang Medical University, Urumqi, Xinjiang Uygur Autonomous Region, China

**Keywords:** disc height, endoscopic foraminoplasty decompression, foraminal area, intraoperative blood loss, lumbar spinal stenosis, spinal canal volume, unilateral biportal endoscopy, nursing care

## Abstract

**Objective:**

To compare perioperative, functional, and radiological outcomes of unilateral biportal endoscopic (UBE) decompression versus endoscopic foraminoplasty decompression (EFD) in patients with single-level lumbar spinal stenosis (LSS) using a retrospective observational design.

**Methods:**

A total of 208 patients with single-level LSS were screened; 161 were enrolled (UBE: *n* = 78; EFD: *n* = 83). Perioperative data, VAS (leg pain) score, ODI, JOA scores, and radiological parameters (disc height, foraminal area, spinal canal volume) were assessed over 12 months. Multivariable regression and repeated-measures ANCOVA were used for adjusted comparisons.

**Results:**

Baseline characteristics were comparable between groups (all *P* > 0.05). UBE had shorter operation time (93.85 vs. 110.42 min, *P* < 0.001) and hospital stay (7.09 vs. 8.71 days, *P* < 0.001), but greater blood loss (143.21 vs. 97.53 mL, *P* < 0.001). Both groups improved significantly in all functional scores (*P* < 0.05). UBE showed faster early recovery at 3–6 months, but differences were non-significant at 12 months. Radiological decompression was more extensive with UBE (*P* < 0.05 for foraminal area and canal volume). Excellent-to-good rates were 66.67% vs. 59.04% (*P* = 0.319). On adjusted analysis, surgical technique was not an independent predictor of favorable outcome (OR = 1.42, 95% CI: 0.71–2.84, *P* = 0.322).

**Conclusion:**

This retrospective comparative observational study suggests that both UBE and EFD are safe and effective minimally invasive options for single-level LSS, with acceptable complication profiles. UBE is associated with shorter operation time and hospital stay but greater blood loss, whereas EFD offers reduced bleeding. Although UBE provides more extensive radiological decompression, functional outcomes at 12 months are comparable between the two techniques. The choice of procedure should be individualized based on stenosis morphology, patient comorbidities, and surgeon expertise. Given the non-randomized design, these findings should be interpreted as hypothesis-generating and require confirmation in prospective randomized trials.

## 1 Introduction

Lumbar spinal stenosis (LSS) is one of the most common degenerative spinal disorders in the aging population. It results from the narrowing of the spinal canal, lateral recess, or intervertebral foramen due to degenerative changes such as hypertrophic ligamentum flavum, osteophyte formation, and disc bulging or herniation (1). This narrowing leads to compression of the cauda equina or nerve roots, manifesting as neurogenic claudication, low back pain, radiating leg pain, and progressive motor or sensory deficits (2, 3). With the global trend of population aging, the prevalence of LSS has risen substantially (4). Epidemiological studies indicate that radiological evidence of LSS can be found in 20%–50% of individuals over 60 years of age, among whom approximately 10%–30% become surgical candidates due to refractory symptoms or neurological deterioration (5).

Conventional surgical treatment, such as posterior lumbar decompression and fusion, effectively relieves neural compression but is associated with considerable surgical trauma, significant blood loss, prolonged hospital stay, and potential complications including adjacent segment degeneration (6). In recent years, minimally invasive endoscopic techniques have revolutionized the management of LSS by offering comparable efficacy with reduced tissue disruption (7). Among these, unilateral biportal endoscopic (UBE) surgery and endoscopic foraminoplasty decompression (EFD) have gained increasing attention (8). UBE employs separate viewing and working portals, allowing independent visualization and instrumentation. This design overcomes the limitations of single-channel endoscopy, permits use of conventional surgical instruments, and facilitates extensive decompression even in complex cases such as central canal stenosis or combined lateral recess and foraminal stenosis (9). In contrast, EFD is a single-channel, fully endoscopic procedure typically performed under local anesthesia. The patient remains conscious during surgery, enabling real-time feedback of neural irritation, thereby improving safety. EFD is particularly advantageous for elderly or medically compromised patients with isolated foraminal stenosis (10, 11).

Despite accumulating evidence supporting the clinical application of both techniques, direct comparative studies between UBE and EFD remain limited, and existing reports show considerable heterogeneity in sample size, follow-up duration, and outcome measures (12, 13). Some authors advocate UBE for its broader decompression capability and superior long-term results, while others emphasize the minimally invasive nature and reduced bleeding of EFD. Moreover, differences in maintaining intervertebral height, expanding foraminal area, and increasing spinal canal volume between the two techniques have not been thoroughly elucidated. Therefore, this retrospective comparative study was designed to compare UBE and EFD in a cohort of 161 patients with LSS, with detailed 12-month follow-up, comprehensive perioperative data, functional scores, and imaging parameters, aiming to provide comparative observational data to inform clinical decision-making.

## 2 Materials and methods

### 2.1 Study design and participants

This was a retrospective comparative study conducted at the First Affiliated Hospital of Xinjiang Medical University. A total of 161 patients with single-level lumbar spinal stenosis who underwent surgery between January 2021 and March 2024 were retrospectively reviewed. Patients were divided into two groups according to the surgical technique they received: the UBE group (*n* = 78) and the EFD group (*n* = 83). No randomization was performed. The study protocol was approved by the Institutional Review Board of the First Affiliated Hospital of Xinjiang Medical University, and all participants provided written informed consent.

### 2.2 Inclusion and exclusion criteria

Inclusion criteria: (1) Diagnosis of lumbar spinal stenosis based on characteristic symptoms (neurogenic claudication, low back pain, leg numbness or weakness), physical findings, and confirmatory imaging (CT or MRI); (2) single-level stenosis; (3) failure of at least 3 months of conservative treatment (medications, physical therapy, rehabilitation); (4) willingness to participate and comply with follow-up.

Exclusion criteria: (1) Concomitant lumbar fracture, spondylolisthesis (grade II or higher), tumor, infection, or other severe spinal disorders; (2) severe cardiopulmonary, hepatic, or renal dysfunction; (3) previous lumbar surgery; (4) psychiatric disorders or cognitive impairment; (5) severe osteoporosis or coagulopathy.

To assess baseline anatomical severity, preoperative lumbar MRI (T2-weighted sagittal and axial) and CT (1-mm slices) were reviewed by two independent radiologists blinded to surgical allocation. Disc height was measured at the midpoint of the affected intervertebral disc on sagittal MRI as the average of anterior, middle, and posterior heights. Ligamentum flavum thickness was measured at the midline on axial MRI at the most stenotic level. Facet joint degeneration was graded according to the Weishaupt classification (grade 0–3) based on axial CT/MRI. Stenosis subtype was categorized as central, lateral recess, foraminal, or mixed based on the predominant compression site. Central canal stenosis severity was further graded using the Schizas classification when applicable. Bone mineral density T-score was obtained from dual-energy x-ray absorptiometry (DEXA) within 6 months preoperatively.

### 2.3 Surgical techniques

The surgical strategy was determined by the attending surgeon based on the predominant type of stenosis and patient-specific factors. Generally, UBE was preferred for patients with central canal stenosis, lateral recess stenosis, or combined stenosis who were fit for general anesthesia. EFD was preferentially selected for patients with isolated foraminal stenosis, advanced age, or high anesthetic risk, as it can be performed safely under local anesthesia. All procedures were performed by two senior spinal surgeons (S.W.B. and S.L.Y.), each with more than 5 years of experience in endoscopic spine surgery and having passed the initial learning curve for both UBE and EFD techniques. The choice of technique was at the surgeon's discretion based on patient-specific factors as described above.

#### 2.3.1 UBE group

UBE was performed under general anesthesia with the patient in the prone position and the abdomen free to reduce intra-abdominal pressure and epidural venous bleeding. Under fluoroscopic guidance, two paramedian skin incisions were made: a 5-mm viewing portal and an 8–10 mm working portal, separated by 2–3 cm. After sequential dilation, normal saline was continuously infused for irrigation. The ligamentum flavum was resected, the spinal canal decompressed, nerve roots mobilized, and herniated disc fragments removed using conventional pituitary rongeurs, Kerrison punches, and burrs. For patients with lateral recess stenosis, the recess was enlarged carefully. At the end of the procedure, the decompression was confirmed by direct visualization, a drain was placed, and the incisions were closed.

#### 2.3.2 EFD group

EFD was performed under local anesthesia with light sedation. The patient was placed prone. Under C-arm guidance, a spinal needle was advanced to the target foramen. A guidewire was introduced, followed by sequential dilators and a working cannula (7–8 mm inner diameter). The endoscope was inserted, and continuous irrigation was applied. Foraminoplasty was performed using endoscopic burrs and reamers to enlarge the foramen. Thereafter, herniated disc tissue, hypertrophic ligamentum flavum, and osteophytes were removed. Throughout the procedure, the patient's lower limb motor and sensory responses were closely monitored. After confirming adequate decompression, the endoscope and instruments were withdrawn, and the incision was closed.

### 2.4 Outcome measures

#### 2.4.1 Perioperative parameters

Operation time (minutes), intraoperative blood loss (mL), 24-hour postoperative drainage volume (mL), postoperative hospital stay (days), and complications (dural tear, surgical site infection, nerve root injury, etc.) were recorded.

#### 2.4.2 Functional outcomes

The Visual Analogue Scale (VAS, 0–10) for leg pain (the predominant symptom in our cohort), Oswestry Disability Index (ODI, 0%–100%), and Japanese Orthopedic Association (JOA, 0–29) score were assessed preoperatively and at 1, 3, 6, and 12 months postoperatively.

#### 2.4.3 Radiological outcomes

**Figure 1:**
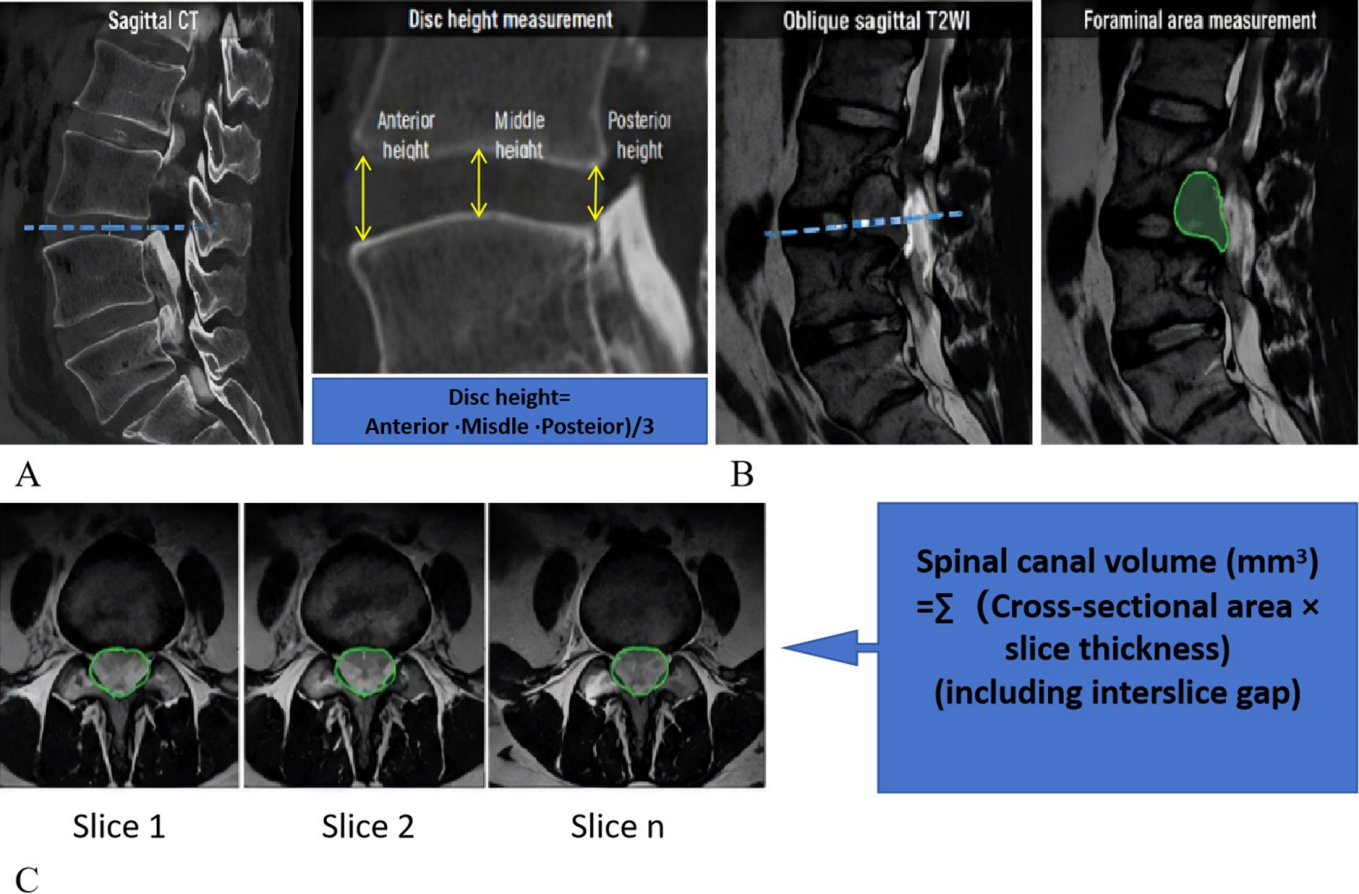
Radiological measurement methodology. (**A**) Sagittal CT and MRI images of the lumbar spine showing the measurement planes for disc height and foraminal area (blue dashed lines). (**B**) Detailed illustration of the disc height measurement formula (anterior, middle, and posterior heights) and the foraminal area outlined in green on oblique sagittal MRI. (**C**) Sequential axial MRI slices with the dural sac cross‑sectional area traced in green, demonstrating the summation method used to calculate spinal canal volume.

All patients underwent standardized lumbar imaging examinations preoperatively and at 1, 3, 6, and 12 months postoperatively, including standing anteroposterior and lateral x-ray, thin-slice computed tomography (CT; 1-mm slice thickness), and magnetic resonance imaging (MRI; 1.5-T or 3.0-T scanner, T2-weighted sagittal and axial sequences). All imaging data were stored and analyzed using the institutional Picture Archiving and Communication System (PACS; Carestream Vue PACS, version 12.1). Two independent spine radiologists, who were blinded to group allocation and clinical outcomes, performed all measurements. The final value for each parameter was calculated as the average of the two observers’ measurements. In case of disagreement >5%, a consensus was reached through joint discussion with a third senior radiologist, see Figure 1A for details.

The following radiological parameters were measured according to standardized protocols:

1. Disc height (mm): Measured on mid-sagittal CT reconstruction images at the center of the affected intervertebral disc. The anterior, middle, and posterior disc heights were measured, and the average of the three values was used for analysis. Preoperative and postoperative disc heights were compared to evaluate maintenance of disc space, see Figure 1B for details.
2. Foraminal area (mm^2^): Measured on oblique sagittal T2-weighted MRI planes parallel to the lumbar foramen at the affected level. The boundaries of the foramen were manually traced using the PACS built-in polygon measurement tool, and the enclosed area was automatically calculated. The foramen was defined by the superior and inferior pedicles, the posterior margin of the vertebral body, and the anterior margin of the superior articular process.
3. Spinal canal volume (mm^3^): Measured on axial T2-weighted MRI images using the sequential summation method. The dural sac cross-sectional area was manually traced at each slice level covering the entire stenotic segment (from the upper endplate of the cephalad vertebra to the lower endplate of the caudad vertebra). The volume was calculated as the sum of cross-sectional area × slice thickness (including interslice gap), expressed in cubic millimeters (mm^3^), see Figure 1C for details. This method has been validated in previous studies for assessing volumetric decompression after endoscopic spine surgery (14, 15).

Interobserver and intraobserver reliability: To assess measurement reproducibility, all parameters were measured independently by the two radiologists. Intraobserver reliability was assessed by having one radiologist repeat the measurements on 20 randomly selected cases after a 2-week interval. The intraclass correlation coefficient (ICC) was calculated using a two-way random-effects model for absolute agreement. ICC values > 0.80 were considered excellent, 0.61–0.80 good, 0.41–0.60 moderate, and < 0.40 poor. In this study, the ICC for interobserver reliability ranged from 0.86 to 0.93 across all parameters, indicating excellent reproducibility. The intraobserver ICC ranged from 0.88 to 0.95.

#### 2.4.4 Clinical efficacy evaluation

At the final follow-up (12 months), the modified MacNab criteria were used to grade outcomes as excellent (symptom-free, normal work/life), good (marked improvement, mild residual symptoms), fair (some improvement but persistent functional limitation), or poor (no change or worsening). The excellent-to-good rate was calculated as (excellent + good)/total × 100%.

### 2.5 Statistical methods

All statistical analyses were performed using SPSS version 26.0 (IBM Corp., Armonk, NY, USA) and R version 4.2.1 (R Foundation for Statistical Computing, Vienna, Austria). Continuous variables were first tested for normality using the Shapiro–Wilk test and for homogeneity of variance using Levene's test. Normally distributed continuous data are presented as mean ± standard deviation (x̅ ±s); non-normally distributed data are presented as median (interquartile range) and were analyzed using non-parametric tests when appropriate. Categorical variables are presented as counts and percentages. Baseline comparisons between the UBE and EFD groups were performed using independent-samples t-tests for normally distributed continuous variables, Mann–Whitney U tests for non-normally distributed continuous variables, and Pearson *χ^2^* tests (or *Fisher's* exact test when expected cell frequencies were <5) for categorical variables.

To account for the non-randomized design and potential confounding, we adopted a multivariable adjustment strategy.

① For perioperative outcomes (operation time, intraoperative blood loss, 24-hour drainage volume, and postoperative hospital stay) and for the 12-month functional and radiological outcomes, we constructed multivariable linear regression models. These models included the following prespecified covariates: age, sex, body mass index (BMI), stenosis subtype (central/lateral recess/foraminal/mixed), preoperative disc height, ligamentum flavum thickness, facet degeneration grade (Weishaupt classification), ASA grade, and the baseline value of the respective outcome variable (e.g., preoperative VAS for the 12-month VAS model, or preoperative foraminal area for the 12-month foraminal area model). Results from these models are presented as adjusted mean differences with 95% confidence intervals (CIs) and corresponding *P* values. ② For longitudinal repeated-measures data (VAS, ODI, and JOA scores, as well as radiological parameters measured at preoperative and 1, 3, 6, and 12 months postoperatively), we performed two-way repeated-measures analysis of covariance (ANCOVA). In these models, the baseline value of the respective outcome (e.g., preoperative VAS for the VAS model) was entered as a covariate to adjust for regression to the mean and to increase statistical precision. The fixed effects included group (UBE vs. EFD), time (5 levels: preoperative, 1, 3, 6, and 12 months), and the group-by-time interaction term, with subject-specific random intercepts. When the sphericity assumption was violated according to Mauchly's test, the Greenhouse-Geisser correction was applied. For each model, we report the *F*-statistics and *P* values for the group effect (overall difference between groups across all time points), the time effect (change over time), and the group-by-time interaction (whether the trajectory of change over time differed significantly between the two groups). For significant interaction terms, *post-hoc* pairwise comparisons were conducted using Bonferroni correction for multiple comparisons, with the number of comparisons explicitly specified for each outcome. ③ For the modified MacNab clinical efficacy grades (ordered categorical: excellent, good, fair, poor) at the final follow-up, comparisons between groups were performed using the Wilcoxon rank-sum test (Mann–Whitney U test). The excellent-to-good rate was additionally compared using the Pearson *χ^2^* test.

To facilitate interpretation of clinical relevance, we calculated effect sizes (*Cohen's d*) for key significant between-group differences and, where applicable, referenced established minimal clinically important differences (MCID) thresholds for VAS (leg pain: 1.5–2.0 points) and ODI (10–15 points) in the Discussion section. All reported *P* values for multiple comparisons are Bonferroni-adjusted *P* values unless otherwise specified. The number of comparisons included in the correction is stated in the footnotes of the corresponding tables.

All tests were two-tailed, and a *P*-value < 0.05 was considered statistically significant. For multivariable regression models, variance inflation factors (VIFs) were calculated to check for multicollinearity, with VIF < 5 considered acceptable.

## 3 Results

### 3.1 Grouping flowchart

**Figure 2:**
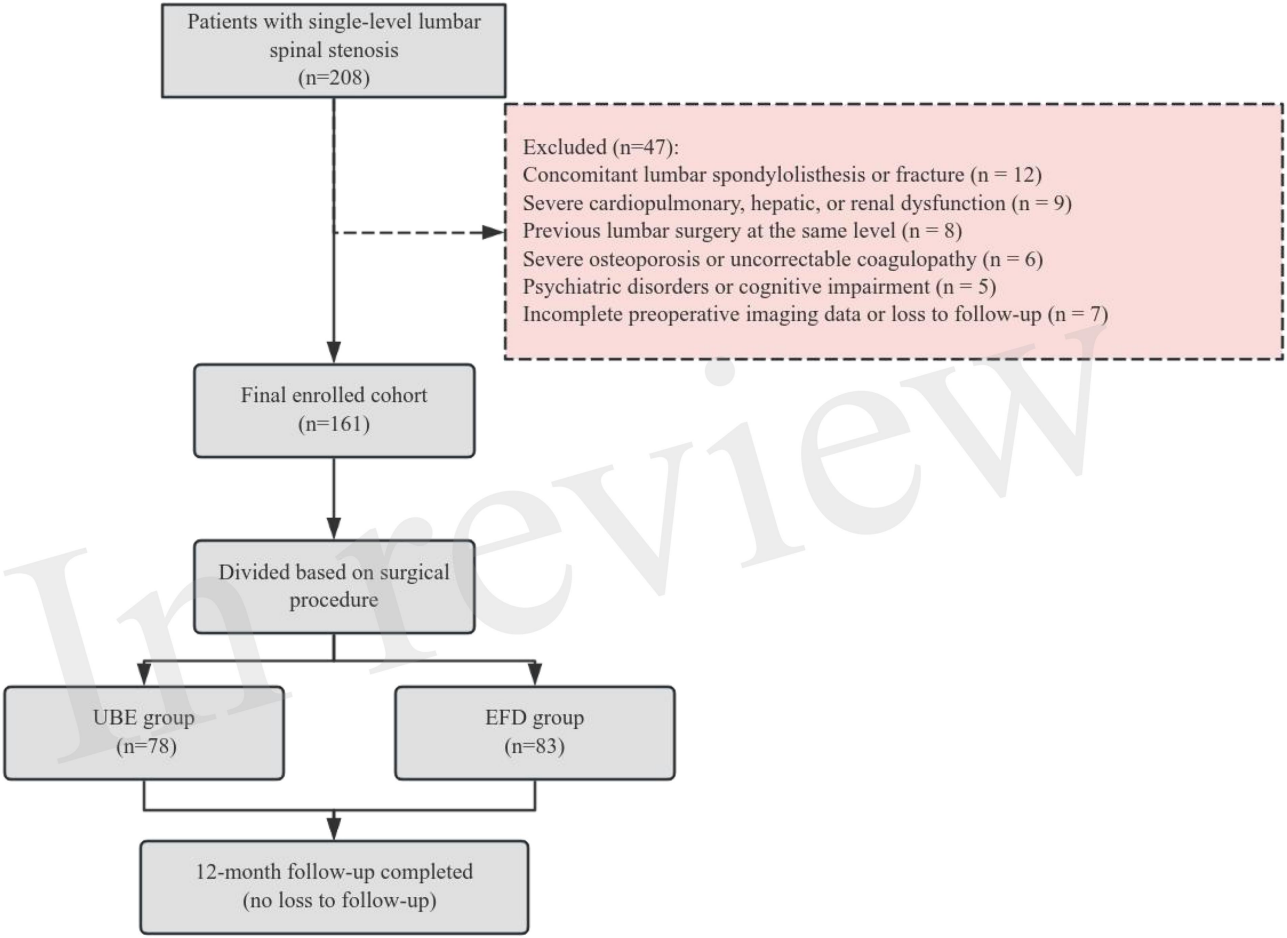
Flow chart of patient assignment.

The study initially screened 208 patients with single-level lumbar spinal stenosis who underwent surgery at our institution between January 2021 and March 2024. After applying the predefined exclusion criteria, 47 patients were excluded: 12 for concomitant lumbar spondylolisthesis (grade ≥Ⅱ) or lumbar fracture; 9 for severe cardiopulmonary, hepatic, or renal dysfunction that precluded safe surgery; 8 for previous lumbar surgery at the same level; 6 for severe osteoporosis (T-score ≤ −3.5) or uncorrectable coagulopathy; 5 for psychiatric disorders or cognitive impairment affecting reliable outcome assessment; and 7 for incomplete preoperative imaging data or loss to follow-up before the 12-month endpoint. Finally, 161 eligible patients were included and allocated to two surgical groups based on the procedure received: 78 underwent UBE and 83 underwent EFD. All enrolled patients completed the 12-month follow-up and were included in the outcome analysis without any additional loss to follow-up. The flowchart detailing the patient selection and group assignment process is presented in Figure 2.

### 3.2 Baseline and perioperative data

There were no significant differences between the two groups in sex, age, body mass index, disease duration, stenosis subtype, preoperative disc height, ligamentum flavum thickness, facet degeneration grade, ASA grade, smoking status, bone mineral density T-score, or level of stenosis (all *P* > 0.05, Table 1). The UBE group had significantly shorter operation time and postoperative hospital stay, but greater intraoperative blood loss and 24-hour drainage volume compared with the EFD group (*P* < 0.001 for all). Complication rates were similar between groups (*P* = 0.683).

**Table 1:** Comparison of perioperative data between the two groups [*n* = 78 (UBE), *n* = 83 (EFD); x̅ ± s or *n* (%)].

Variable	UBE group (*n* = 78)	EFD group (*n* = 83)	*t/χ^2^/Z*	*P*
Sex			0.136	0.713
Male	50 (64.10)	55 (66.27)		
Female	28 (35.90)	28 (33.73)		
Age (years)	58.63 ± 10.82	58.34 ± 10.59	0.172	0.864
Body mass index (kg/m^2^)	22.18 ± 1.45	22.40 ± 1.51	−0.952	0.343
Disease duration (years)	7.14 ± 1.60	7.28 ± 1.53	−0.568	0.571
Stenosis subtype			0.547	0.460
Central	20 (25.64)	16 (19.28)		
Lateral recess	23 (29.49)	27 (32.53)		
Foraminal	16 (20.51)	30 (36.14)		
Mixed (central + lateral/foraminal)	19 (24.36)	10 (12.05)		
Schizas grade (for central stenosis, *n* = 39 UBE, *n* = 26 EFD)			0.918	0.338
Grade A–B	14 (35.90)	12 (46.15)		
Grade C–D	25 (64.10)	14 (53.85)		
Preoperative disc height (mm)	9.31 ± 0.85	9.42 ± 0.79	−0.734	0.464
Ligamentum flavum thickness (mm)	4.76 ± 1.18	4.91 ± 1.24	−0.789	0.431
Facet joint degeneration grade (Weishaupt)			0.341	0.559
Grade 0–1	28 (35.90)	32 (38.55)		
Grade 2–3	50 (64.10)	51 (61.45)		
ASA physical status			0.215	0.643
I–II	60 (76.92)	66 (79.52)		
III–IV	18 (23.08)	17 (20.48)		
Smoking (current)	25 (32.05)	28 (33.73)	0.083	0.773
Bone mineral density T-score	−1.52 ± 0.98	−1.63 ± 1.05	0.695	0.488
Level of stenosis			0.347	0.841
L3-4	10 (12.82)	11 (13.25)		
L4-5	34 (43.59)	34 (40.96)		
L5-S1	34 (43.59)	38 (45.78)		
Operation time (min)	93.85 ± 14.62	110.42 ± 18.11	−6.397	<0.001
Intraoperative blood loss (mL)	143.21 ± 32.08	97.53 ± 19.64	11.087	<0.001
24 h postoperative drainage (mL)	92.18 ± 16.45	68.95 ± 9.32	11.373	<0.001
Postoperative hospital stay (days)	7.09 ± 1.58	8.71 ± 2.13	−5.389	<0.001
Complications (Total)	4 (5.13)	7 (8.43)	0.166	0.683
Dural tear	2 (2.56)	2 (2.41)		
Surgical site infection	1 (1.28)	3 (3.61)		
Nerve root injury	1 (1.28)	2 (2.41)		

Data for stenosis subtype, Schizas grade, disc height, ligamentum flavum thickness, facet grade, ASA grade, smoking, and BMD T-score were retrospectively collected from preoperative imaging and medical records. All continuous variables were compared using independent-samples t-tests; categorical variables were compared using Pearson χ^2^ tests. No significant baseline differences were observed between the two groups for any of the newly added variables (all *P* > 0.05).

### 3.3 Functional outcomes

**Figure 3:**
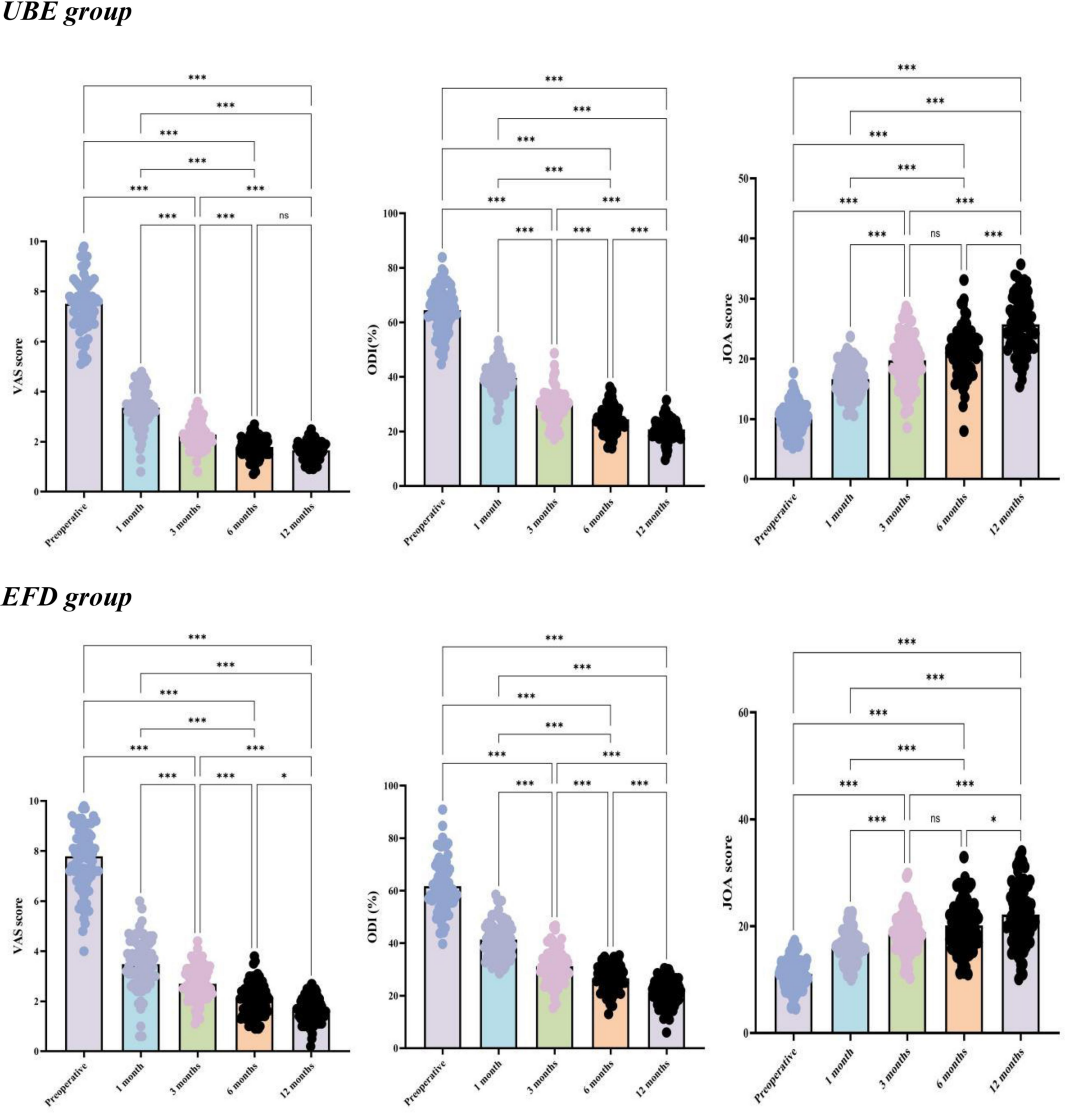
Comparison of clinical outcome scores between the two groups. *, *** There's a statistical difference between the two groups.

Repeated-measures ANOVA revealed significant improvements in VAS, ODI, and JOA scores over time in both groups (all *P* < 0.001 for time effect). The UBE group had significantly lower VAS scores at 3 and 6 months postoperatively (*P* = 0.002 and *P* = 0.005, respectively), and lower ODI scores and higher JOA scores at 6 months (*P* = 0.001 and *P* = 0.003, respectively). By 12 months, differences between groups were no longer statistically significant (*P* > 0.05). Detailed data are presented in Table 2 and Figure 3.

**Table 2:** Comparison of clinical outcome scores between the two groups.

Variable	Time point	UBE group (*n* = 78)	EFD group (*n* = 83)	Mean difference (95% CI)	*P* value*	Cohen's *d*
VAS score (leg pain)						
	Preoperative	7.49 ± 1.28	7.60 ± 1.33	−0.11 (−0.52 to 0.30)	0.589	0.08
	1 month	3.35 ± 0.81**	3.50 ± 1.04**	−0.15 (−0.44 to 0.14)	0.309	0.16
	3 months	2.45 ± 0.59**	2.78 ± 0.73**	**−0.33 (−0.54 to −0.12)**	**0**.**002**	**0**.**50**
	6 months	1.76 ± 0.48**	2.00 ± 0.59**	**−0.24 (−0.41 to −0.07)**	**0**.**005**	**0**.**45**
	12 months	1.58 ± 0.41**	1.67 ± 0.50**	−0.09 (−0.23 to 0.05)	0.220	0.20
ODI (%)						
	Preoperative	65.38 ± 8.92	65.03 ± 9.10	0.35 (−2.45 to 3.15)	0.804	0.04
	1 month	39.95 ± 6.52**	41.77 ± 7.20**	−1.82 (−3.94 to 0.30)	0.091	0.27
	3 months	29.62 ± 5.68**	31.68 ± 6.25**	**−2.06 (−3.93 to −0.19)**	**0**.**031**	**0**.**35**
	6 months	24.41 ± 4.80**	26.88 ± 5.20**	**−2.47 (−4.01 to −0.93)**	**0**.**001**	**0**.**50**
	12 months	20.75 ± 4.18**	21.88 ± 4.70**	−1.13 (−2.52 to 0.26)	0.111	0.25
JOA score						
	Preoperative	10.32 ± 2.65	10.68 ± 2.78	−0.36 (−1.20 to 0.48)	0.395	0.13
	1 month	16.68 ± 3.20**	16.01 ± 3.37**	0.67 (−0.34 to 1.68)	0.195	0.20
	3 months	19.82 ± 3.71**	18.71 ± 3.95**	1.11 (−0.08 to 2.30)	0.067	0.29
	6 months	22.41 ± 4.15**	20.78 ± 4.38**	**1.63 (0.31 to 2.95)**	**0**.**003**	**0**.**39**
	12 months	24.32 ± 4.66**	23.35 ± 4.97**	0.97 (−0.51 to 2.45)	0.199	0.20

Mean Difference = UBE group mean minus EFD group mean; a negative value for VAS and ODI favors UBE; a positive value for JOA favors UBE.

Cohen's *d* ≥0.2 indicates small effect, ≥0.5 moderate effect, ≥0.8 large effect (interpreted cautiously).

* *P* values shown are Bonferroni-adjusted for 5 time-point comparisons per outcome variable (adjusted *α*= 0.05/5 = 0.010 per individual comparison). Values in bold indicate statistically significant between-group differences after Bonferroni correction.

** *P* < 0.05 compared with preoperative value within the same group (paired t-test).

Repeated-measures ANCOVA revealed significant improvements in VAS, ODI, and JOA scores over time in both groups (all *P* < 0.001 for time effect). The group-by-time interaction was significant for VAS scores (*F* = 3.42, *P* = 0.018) and ODI scores (*F* = 3.01, *P* = 0.031) at the 3- and 6-month time points, indicating that the UBE group experienced faster early improvement. However, the interaction was no longer significant at 12 months for VAS (*F* = 1.21, *P* = 0.214), ODI (*F* = 0.98, *P* = 0.325), or JOA (*F* = 1.15, *P* = 0.284), supporting the convergence of functional outcomes between the two groups by the final follow-up.

### 3.4 Radiological outcomes

Both groups demonstrated significant increases in disc height, foraminal area, and spinal canal volume after surgery (*P* < 0.001 for time effect). The UBE group showed greater improvements in most radiological parameters, with statistically significant differences at 3 months for disc height (*P* = 0.042), at 1, 3, 6, and 12 months for foraminal area (all *P* < 0.05), and at 3, 6, 12 months for spinal canal volume (all *P* < 0.05). The results are summarized in Table 3.

**Table 3:** Comparison of radiological parameters between the two groups.

Variable	Time point	UBE group (*n* = 78)	EFD group (*n* = 83)	Mean difference (95% CI)	*P* value*	Cohen's *d*
Disc height (mm)						
	Preoperative	9.55 ± 0.70	9.62 ± 0.62	−0.07 (−0.27 to 0.13)	0.499	0.11
	1 month	10.09 ± 0.86**	9.86 ± 0.68**	0.23 (−0.01 to 0.47)	0.062	0.30
	3 months	10.25 ± 1.08**	9.94 ± 0.85**	**0.31 (0.01 to 0.61)**	**0**.**042**	**0**.**32**
	6 months	10.38 ± 1.25**	10.03 ± 1.09**	0.35 (−0.01 to 0.71)	0.057	0.30
	12 months	10.60 ± 1.22**	10.26 ± 1.14**	0.34 (−0.02 to 0.70)	0.066	0.29
Foraminal area (mm^2^)						
	Preoperative	80.34 ± 10.95	81.16 ± 11.20	−0.82 (−4.26 to 2.62)	0.638	0.07
	1 month	95.98 ± 12.52**	90.32 ± 11.35**	**5.66 (1.95 to 9.37)**	**0**.**003**	**0**.**48**
	3 months	98.62 ± 13.38**	92.27 ± 12.28**	**6.35 (2.36 to 10.34)**	**0**.**002**	**0**.**50**
	6 months	100.81 ± 14.50**	95.44 ± 13.35**	**5.37 (1.05 to 9.69)**	**0**.**015**	**0**.**39**
	12 months	102.99 ± 15.02**	98.49 ± 14.28**	**4.50 (0.02 to 8.98)**	**0**.**049**	**0**.**31**
Spinal canal volume (mm^3^)						
	Preoperative	1192.5 ± 154.3	1204.8 ± 159.6	−12.3 (−60.1 to 35.5)	0.615	0.08
	1 month	1405.3 ± 185.2**	1352.6 ± 174.1**	52.7 (−2.8 to 108.2)	0.064	0.29
	3 months	1455.8 ± 195.0**	1384.2 ± 184.0**	**71.6 (12.8 to 130.4)**	**0**.**017**	**0**.**38**
	6 months	1507.4 ± 206.5**	1426.3 ± 195.5**	**81.1 (18.5 to 143.7)**	**0**.**011**	**0**.**40**
	12 months	1558.2 ± 215.8**	1459.8 ± 206.1**	**98.4 (33.5 to 163.3)**	**0**.**003**	**0**.**47**

Mean Difference = UBE group mean minus EFD group mean; a positive value for all parameters favors UBE.

Cohen's *d* ≥0.2 indicates small effect, ≥0.5 moderate effect, ≥0.8 large effect (interpreted cautiously).

* *P* values shown are Bonferroni-adjusted for 5 time-point comparisons per outcome variable (adjusted *α*= 0.05/5 = 0.010 per individual comparison). Values in bold indicate statistically significant between-group differences after Bonferroni correction.

** *P* < 0.05 compared with preoperative value within the same group (paired t-test).

For radiological parameters, the group-by-time interaction was significant for foraminal area (*F* = 4.12, *P* = 0.008) and spinal canal volume (*F* = 3.89, *P* = 0.012) across the entire study period, reflecting the consistently greater radiographic decompression achieved in the UBE group. By contrast, the interaction for disc height did not reach statistical significance (*F* = 1.67, *P* = 0.158), suggesting that the between-group differences in disc height restoration were less pronounced over time.

### 3.5 Complications and clinical efficacy

#### 3.5.1 Complications

A detailed breakdown of complications is presented in Table 4. The overall complication rate was 5.13% (4/78) in the UBE group and 4.82% (4/83) in the EFD group, with no significant difference between groups (*P* = 1.000). Dural tears occurred in 2 patients (2.56%) in the UBE group and 2 patients (2.41%) in the EFD group; all were minor (<5 mm) and managed successfully with fibrin glue application or primary suture without sequelae. Nerve root injury occurred in 1 UBE patient (1.28%) and 2 EFD patients (2.41%); all cases presented as transient postoperative weakness and recovered completely with conservative management within 3 months. One patient (1.28%) in the UBE group developed a small epidural hematoma, which was managed conservatively without neurological deterioration. No patient in either group required reoperation within 30 days, and none required conversion to open surgery. Readmission within 30 days occurred in 1 UBE patient (1.28%) for urinary tract infection and in 2 EFD patients (2.41%) for wound-related issues; all were managed conservatively and discharged uneventfully. According to the Clavien–Dindo classification, all complications were Grade I or Grade II, with no Grade III or IV complications in either group.

**Table 4:** Detailed comparison of perioperative complications between the two groups.

Complication	UBE group (*n* = 78)	EFD group (*n* = 83)	Clavien–Dindo grade	Management	*P* value
Intraoperative complications					
Dural tear	2 (2.56)	2 (2.41)	Grade I	Fibrin glue/primary suture	1.000*
Nerve root injury	1 (1.28)	2 (2.41)	Grade I	Conservative (observation)	1.000*
Postoperative complications					
Epidural hematoma	1 (1.28)	0 (0)	Grade I	Conservative (observation)	0.484*
Reoperation and readmission					
Reoperation within 30 days	0 (0)	0 (0)	—	—	—
Readmission within 30 days	1 (1.28)	2 (2.41)	Grade II	Conservative (UTI/wound care)	1.000*
Conversion to open surgery	0 (0)	0 (0)	—	—	—
Total complications	4 (5.13)	4 (4.82)	—	—	1.000**

Values are presented as *n* (%).

UTI, urinary tract infection.

Clavien–Dindo classification: Grade I = deviation from normal postoperative course without pharmacological treatment or surgical/radiological intervention; Grade II = requiring pharmacological treatment; Grade III = requiring surgical, endoscopic, or radiological intervention; Grade IV = life-threatening complication requiring ICU management. No Grade III or IV complications occurred in either group.

* Individual complication types were compared using Fisher's exact test; no significant differences were found for any specific type.

** Overall complication rate was compared using Pearson χ^2^ test.

#### 3.5.2 Clinical efficacy according to modified MacNab criteria

**Figure 4:**
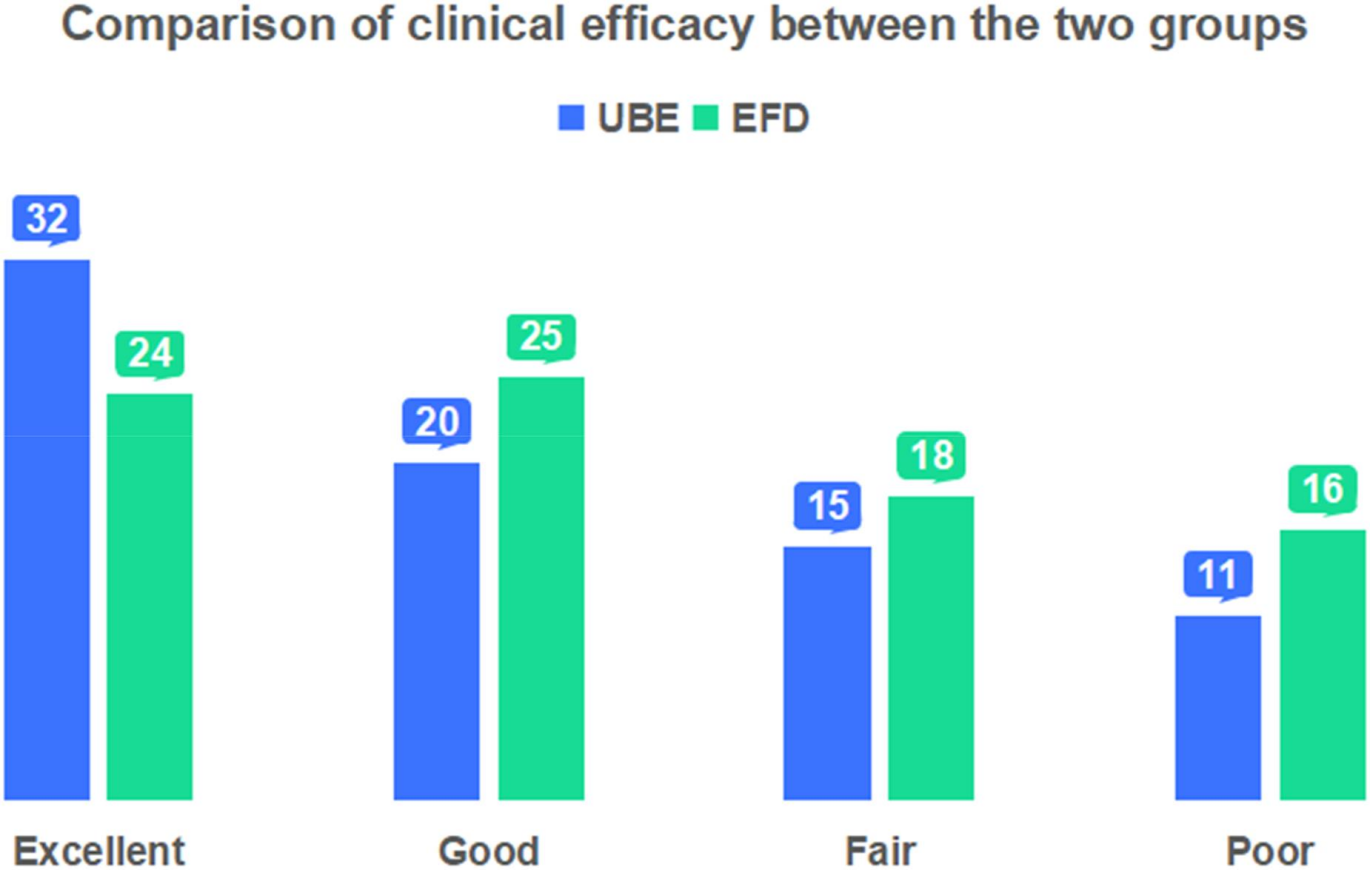
Comparison of clinical efficacy between the two groups.

At the final follow-up (12 months), the distribution of outcomes according to the modified MacNab criteria is shown in Table 5 and Figure 4. The UBE group had a higher proportion of excellent results (41.03% vs. 28.92%) and a higher excellent-to-good rate (66.67% vs. 59.04%), but the difference did not reach statistical significance (*P* = 0.287 for rank distribution; *P* = 0.319 for excellent-to-good rate). On multivariable logistic regression analysis adjusting for age, sex, BMI, stenosis subtype, baseline ODI, and surgical technique, the only independent predictor of achieving an excellent or good outcome at 12 months was baseline ODI severity (adjusted OR = 0.94 per 1-point increase, 95% CI: 0.90–0.98, *P* = 0.006), indicating that patients with milder preoperative disability had higher odds of favorable outcome regardless of the surgical technique received. Surgical technique itself was not an independent predictor (adjusted OR = 1.42, 95% CI: 0.71–2.84, *P* = 0.322).

**Table 5:** Comparison of clinical efficacy between the two groups [*n* (%)].

Group	*n*	Excellent	Good	Fair	Poor	Excellent-to-good rate
UBE	78	32 (41.03)	20 (25.64)	15 (19.23)	11 (14.10)	52 (66.67)
EFD	83	24 (28.92)	25 (30.12)	18 (21.69)	16 (19.28)	49 (59.04)
*Z/χ^2^*			1.065			0.995
*P*			0.287			0.319

### 3.6 Typical cases

A 58-year-old male presented with low back pain and right lower limb radiating pain for 3 months and intermittent claudication after approximately 20 meters. MRI confirmed L4/5 lumbar spinal stenosis. After unilateral biportal endoscopic (UBE) decompression, the right lower limb pain was significantly relieved, and the patient was able to ambulate with a lumbar brace on the first postoperative day, with resolution of claudication.

**Figure 5:**
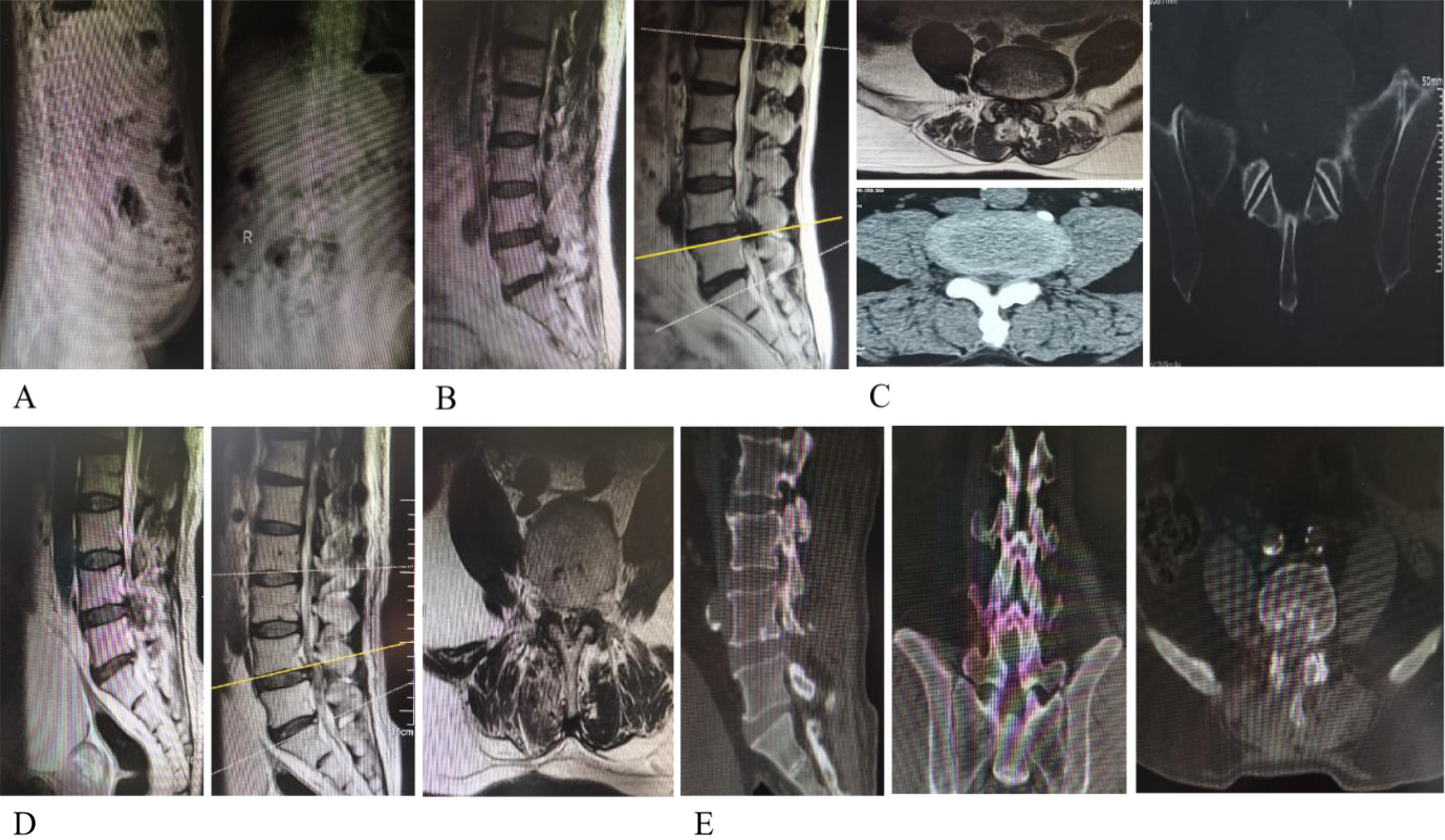
Preoperative and postoperative imaging of a representative patient in the UBE group (L4/5 level). (**A**) Preoperative lateral X‑ray showing straightening of lumbar lordosis, mild reduction of disc height at L4/5, and marginal osteophytes, without spondylolisthesis or fracture. (**B**) Preoperative T2‑weighted sagittal MRI demonstrating marked ligamentum flavum hypertrophy, dural sac compression, severe lateral recess narrowing, and loss of cerebrospinal fluid signal around the right nerve root. (**C**) Preoperative axial CT image revealing hypertrophic and inwardly displaced facet joints, shortened sagittal canal diameter, and osseous lateral recess stenosis, with no obvious posterior longitudinal ligament ossification. (**D**) Postoperative T2‑weighted sagittal MRI showing adequate decompression of the spinal canal, restoration of the dural sac contour, and improved cerebrospinal fluid signal around the nerve roots. (**E**) is the postoperative CT.

Figure 5A is the preoperative X-ray, showing straightening of the lumbar lordosis, mild reduction of the L4/5 disc height, and osteophyte formation at the anterior and posterior margins of the vertebral bodies, without evidence of lumbar spondylolisthesis or fracture. Figure 5B is the preoperative MRI, showing marked hypertrophy of the ligamentum flavum at L4/5, compression and deformation of the dural sac, significant reduction of the lateral recess and spinal canal effective volume, displacement of the right nerve root, and loss of cerebrospinal fluid signal around the nerve root on T2-weighted images. Figure 5C is the preoperative CT, showing hypertrophic and inwardly displaced facet joints at L4/5, shortened sagittal diameter of the spinal canal, osseous lateral recess stenosis, and no obvious ossification of the posterior longitudinal ligament at the posterior vertebral margin. Figure 5D is the postoperative MRI, showing adequate decompression of the L4/5 spinal canal, good restoration of dural sac morphology, patent cerebrospinal fluid signal, reappearance of the perineural space around the right nerve root, and no residual compression or postoperative hematoma. Figure 5E is the postoperative CT.

A 55-year-old female presented with low back pain and right lower limb radiating pain for 6 years. MRI confirmed L4/5 lumbar spinal stenosis. After endoscopic foraminoplasty decompression (EFD), the right lower limb pain was significantly relieved, and the patient was able to ambulate with a lumbar brace on the first postoperative day, with resolution of claudication.

**Figure 6:**
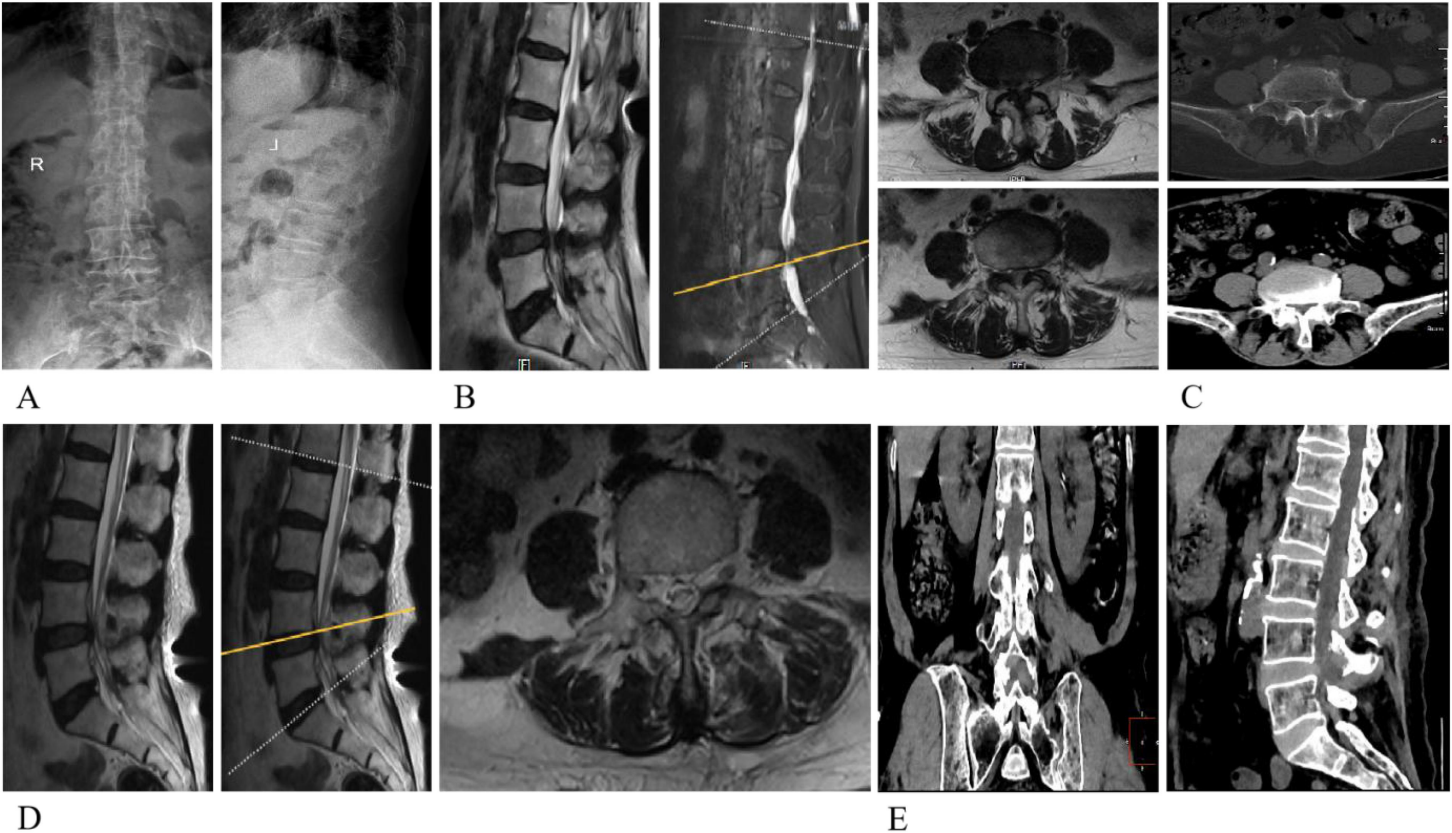
Preoperative and postoperative imaging of a representative patient in the EFD group (L4/5 level). (**A**) Preoperative lateral X‑ray showing loss of disc height and marginal osteophyte formation at L4/5, without instability or fracture. (**B**) Preoperative T2‑weighted sagittal MRI revealing severe foraminal stenosis at L4/5 with nerve root compression and loss of surrounding cerebrospinal fluid signal. (**C**) Preoperative axial CT image demonstrating hypertrophic facet joints and narrowing of the intervertebral foramen, with no significant ossification of the posterior longitudinal ligament. (**D**) Postoperative T2‑weighted sagittal MRI showing successful foraminal decompression, improved nerve root passage, and restored cerebrospinal fluid signal around the exiting nerve root.

Figure 6A is the preoperative X-ray, showing slight straightening of the lumbar lordosis, mild loss of L4/5 disc height, and mild osteophyte formation at the anterior and posterior vertebral margins, without evidence of lumbar spondylolisthesis or vertebral fracture. Figure 6B is the preoperative MRI, showing decreased signal intensity of the L4/5 disc with posterior bulging, hypertrophic ligamentum flavum, marked narrowing of the right foramen, compression of the right L4 nerve root, deformed dural sac, and disruption of cerebrospinal fluid signal at the corresponding level. Axial views show significant reduction of the lateral recess and foraminal effective area. Figure 6C is the preoperative CT, showing hypertrophic and inwardly displaced facet joints at L4/5 with osseous foraminal stenosis on the right, no obvious ossification of the posterior longitudinal ligament, but dense bony structures at the entrance of the foramen, consistent with degenerative foraminal stenosis. Figure 6D is the postoperative MRI. Figure 6E is the postoperative CT, showing adequate resection of the posterior margin of the right foramen and part of the facet joint, enlargement of the osseous canal, thorough decompression of the spinal canal and lateral recess, stable bony structures, and no obvious destruction of adjacent vertebrae.

## 4 Discussion

In this retrospective cohort study, we compared the perioperative, functional, and radiological outcomes of UBE versus EFD in patients with single-level lumbar spinal stenosis. Both techniques proved to be safe and effective, with significant improvements in pain, disability, and imaging parameters lasting at least 12 months. However, each technique exhibited distinct advantages and drawbacks that may guide surgical decision-making.

The UBE group had a significantly shorter operation time and postoperative hospital stay, which is consistent with previous reports (16). The dual-channel design allows independent use of a full-sized endoscope and conventional surgical instruments without mutual interference. This setup facilitates faster bone removal and ligament resection, especially when using Kerrison punches or burrs of larger diameter (17–19). These findings are in line with recent comparative studies. Li et al. also reported shorter operation times for UBE compared to one-hole split endoscopy, a uniportal technique. Similarly, Heo et al. found that UBE was more efficient than uniportal endoscopy for central stenosis, largely due to the superior maneuverability and visualization (12, 14). In contrast, EFD requires frequent exchange of instruments through a single working channel, prolonging the operation. Notably, despite longer operative time, EFD resulted in significantly less intraoperative blood loss and lower 24-hour drainage volume. This can be attributed to the precise transforaminal approach under local anesthesia, which minimizes paravertebral muscle injury and avoids major epidural veins. Additionally, the continuous irrigation under pressure may contribute to hemostasis (14). This advantage is particularly important for elderly or frail patients. As demonstrated by Houra et al. in their long-term study, EFD is a safe and effective option for single-level foraminal stenosis, with minimal blood loss and the benefit of being performed under local anesthesia, which is a major advantage over UBE in medically compromised patients (10).

Complication rates did not differ significantly between groups. Dural tears occurred in 2.56% of UBE and 2.41% of EFD patients, all of which were minor (<5 mm) and managed successfully with fibrin glue or sutures. Postoperative infections were slightly more frequent in the EFD group (3.61% vs. 1.28%), possibly due to repeated instrument passage through the same portal, increasing soft tissue trauma and bacterial exposure. Nerve root injuries were rare in both groups, and all patients recovered with conservative management (20–22).

Regarding functional recovery, both groups showed progressive improvement in VAS, ODI, and JOA scores. The UBE group demonstrated significantly better pain relief and functional outcomes at 3 and 6 months postoperatively, but these differences disappeared by 12 months. This intriguing finding suggests that although EFD provides less extensive immediate decompression, the comparable functional outcomes at 12 months may be attributed to the gradual resolution of postoperative nerve root edema and inflammatory cytokines, as well as the spinal cord's intrinsic adaptive capacity to residual compression, provided that the residual stenosis remains below a critical symptomatic threshold. Furthermore, the extended 12-month recovery period allows for maximal neuromuscular rehabilitation, which may offset the initial anatomical differences (23–25). This phenomenon aligns with the findings of several comparative studies. Sun et al. observed that while UBE offered superior early functional recovery compared to percutaneous endoscopic discectomy for foraminal lesions, the differences diminished over time, indicating a convergence of clinical outcomes regardless of the initial radiological decompression extent (7).

Although some between-group differences in VAS and ODI reached statistical significance, the magnitude of these differences fell below the established minimal clinically important differences (MCID) for these instruments (VAS: 1.5–2.0 points; ODI: 10–15 points). For instance, the mean ODI difference at 6 months was only 2.47 points (95% CI: 0.93–4.01). This suggests that the superior radiological decompression achieved by UBE did not translate into clinically meaningful functional superiority at 12 months.

From a radiological perspective, UBE achieved superior restoration of disc height, foraminal area, and spinal canal volume at all postoperative time points. The greater decompression capacity of UBE likely results from better preservation of the facet joints and paraspinal musculature, as the dual portals allow a more tailored resection of the hypertrophic ligamentum flavum and osteophytes while maintaining spinal stability (26, 27). In contrast, EFD, being a single-portal technique, inherently limits the working angle and the extent of bone resection. Moreover, repeated repositioning of the working cannula during EFD may cause cumulative microtrauma to the foraminal soft tissues, potentially leading to slight loss of foraminal height over time. Consistent with our results, Pao (26) reported significant increases in both foraminal area and spinal canal volume post-UBE, highlighting its powerful and precise decompression capability (26). Nevertheless, the clinical relevance of these radiological differences remains debatable, as the 12-month functional scores were equivalent between groups (28–30).

This retrospective study has several strengths, including a relatively large sample size (*n* = 161), complete 12-month follow-up, multidimensional outcome assessment with blinded radiologists, and rigorous statistical analysis using repeated-measures ANOVA with Bonferroni correction. Nevertheless, several limitations should be acknowledged. The sample size was insufficient for subgroup analyses by stenosis type. The 12-month follow-up cannot address long-term durability or adjacent segment degeneration. Being a single-center study with experienced surgeons, generalizability may be limited, especially for EFD, which has been suggested to have a more demanding learning phase based on institutional experience and recent literature. Complete blinding was infeasible due to differences in incision number and anesthesia type. Cost-effectiveness was not analyzed. Patients with multilevel stenosis, high-grade spondylolisthesis, or severe osteoporosis were excluded, limiting applicability to complex cases. Radiological measurements may have inter-observer variability (kappa not calculated). The lack of a conservative treatment control group prevents comparison with natural history. Finally, the modified MacNab criteria are somewhat subjective; future studies should include objective gait analysis or PROMs. Despite these limitations, both UBE and EFD are effective minimally invasive options for LSS. UBE is preferable for central or mixed stenosis requiring extensive decompression due to shorter operative time and superior radiological restoration. EFD is advantageous for elderly, frail, or high-bleeding-risk patients with isolated foraminal stenosis, offering minimal blood loss and local anesthesia. Future multicenter randomized trials with longer follow-up, inclusion of complex stenosis patterns, advanced imaging, and cost-utility analyses are warranted. Elucidating the molecular mechanisms of delayed functional equivalence may facilitate adjunctive therapies to accelerate recovery. Fourth, the surgical technique was not randomized; therefore, selection bias is inherent. Although we reported the selection preferences, residual confounding by stenosis severity and surgeon decision-making cannot be entirely excluded. We acknowledge that despite the inclusion of multiple additional baseline variables (stenosis subtype, disc height, ligamentum flavum thickness, facet grade, ASA, smoking, BMD), some unmeasured confounders such as detailed sagittal alignment parameters and preoperative physical activity level may still have influenced outcomes. Fifth, although all procedures were performed by experienced surgeons who had passed the learning curve for both techniques, the single-center design and the operators’ familiarity with each technique may limit generalizability. Surgeon experience and institutional volume may influence outcomes, particularly for EFD, which has been reported to have a more demanding learning phase. However, we did not directly measure learning curve effects in this study. Additionally, UBE and EFD differed not only in the surgical approach but also in anesthesia type (general vs. local). This inherent difference may have independently influenced operative time, fluid management, and postoperative mobilization, thereby affecting hospital stay and early recovery parameters. Thus, the perioperative benefits cannot be entirely attributed to the surgical technique alone.

## 5 Conclusion

Both UBE and EFD are safe and effective surgical treatments for lumbar spinal stenosis. UBE is associated with shorter operation time and hospital stay but greater intraoperative blood loss, whereas EFD offers reduced bleeding at the expense of longer operative time and potentially a more demanding learning phase, which should be considered during adoption of the technique. Radiological decompression is more extensive after UBE, yet clinical outcomes at 12 months are comparable. Patient-specific factors and surgeon experience should guide the selection of the optimal technique.

## Data Availability

The raw data supporting the conclusions of this article will be made available by the authors, without undue reservation.

## References

[1] Ogihara A Fukuda T Katsumi S Ojiri H. Diagnostic performance of CT-like images for lumbar pedicle screw planning and spinal canal area measurement: a comparative study with conventional CT and MRI. Tomography. (2026) 12(3):40. 10.3390/tomography12030040 41893835 PMC13030672

[2] Huang R Pan F Zhu W Kong C Lu S. Predictors for the restoration of the sagittal spinal malalignment in patients with lumbar stenosis after short-segment decompression and fusion surgery. BMC Musculoskel Dis. (2022) 23(1):711. 10.1186/s12891-022-05666-2 PMC9316683 35883059

[3] Zhang H Dong Z Liu W Pu F Cao J Du S. Clinical efficacy and safety of arthroscopic-assisted uniportal spinal surgery versus percutaneous endoscopic lumbar decompression in lumbar spinal stenosis: a retrospective study. Orthop Surg. (2026) 18(2):261–9. 10.1111/os.70237 41491889 PMC12862444

[4] Chen M Zhang P Lai J Li S Yu W Fan S. A correlation study of preoperative lumbar paraspinal muscle quality and L5-S1 lumbar foraminal stenosis degeneration after L4-5 TLIF. J Orthop Surg Res. (2023) 18(1):731. 10.1186/s13018-023-04196-4 37752600 PMC10523737

[5] Cui H Zhou M Gong Y Zhang H Zhang T Tan X. Impact of different paraspinal muscle mass on the prognosis of ACDF-a finite element analysis. Front Bioeng Biotech. (2026) 14:1743924. 10.3389/fbioe.2026.1743924 PMC12968279 41808768

[6] Shang Q Luan H Peng C Song X. Comparative effectiveness of cortical bone trajectory screws and pedicle screws in the treatment of adjacent segment degeneration after lumbar fusion surgery: a systematic review and meta-analysis. J Orthop Surg Res. (2024) 19(1):380. 10.1186/s13018-024-04865-y 38943143 PMC11212258

[7] Sun Y Tian D Chen L Zhu B Zhong H Jing J. Comparison of unilateral biportal and percutaneous endoscopic discectomy in treating far lateral lumbar disc herniation. Joint Dis Relat Surg. (2026) 37(1):16–26. 10.52312/jdrs.2026.2326 PMC12788751 41391115

[8] Haibier A Wang J Liu W Wang G. Comprehensive identification of risk factors for recurrence after percutaneous endoscopic lumbar discectomy: a systematic review and meta-analysis. Front Surg. (2026) 13:1733796. 10.3389/fsurg.2026.1733796 41953493 PMC13055515

[9] Yuan S Wang A Fan N Du P Wang T Li J. Recompression after percutaneous transforaminal endoscopic decompression for degenerative lumbar spinal stenosis: risk factors and outcomes of two different reoperation procedures. Front Surg. (2024) 11:1392215. 10.3389/fsurg.2024.1392215 38978988 PMC11228264

[10] Houra K Saftic R Klaric E Knight M. Long-term clinical outcomes following endoscopic foraminoplasty for patients with single-level foraminal stenosis of the lumbar spine. Int J Spine Surg. (2022) 16(1):139–50. 10.14444/8182 35177520 PMC9519075

[11] Wang X Zhang A Yao W Qiu H Feng F. Bibliometric analysis of transforaminal lumbar interbody fusion: research status, trends, and future directions. Efort Open Rev. (2023) 8(12):906–18. 10.1530/EOR-23-0155 38038386 PMC10714383

[12] Li T Jiang Q Zhong W Zhu T Lu Z Ding Y. One-hole split endoscope versus unilateral biportal endoscopy for lumbar spinal stenosis: a retrospective propensity score study. J Orthop Surg Res. (2024) 19(1):254. 10.1186/s13018-024-04743-7 38649974 PMC11034078

[13] Ren Y Yu T Qiu X Chen W Zhang W Zhao H. [Comparison of clinical efficacy of unilateral dual-channel endoscopy and large-channel endoscopy techniques in the treatment of lumbar spinal canal stenosis]. Zhongguo Gu Shang. (2026) 39(3):246–53. 10.12200/j.issn.1003-0034.20240650 41930384

[14] Heo DH Lee DC Park CK. Comparative analysis of three types of minimally invasive decompressive surgery for lumbar central stenosis: biportal endoscopy, uniportal endoscopy, and microsurgery. Neurosurg Focus. (2019) 46(5):E9. 10.3171/2019.2.FOCUS197 31042664

[15] Mizouchi T Watanabe K Izumi T Katsumi K Shoji H Ohashi M. Quantitative radiographic analysis of foraminal re-stenosis after posterior cervical foraminotomy with laminoplasty. J Clin Neurosci. (2019) 67:99–104. 10.1016/j.jocn.2019.06.012 31227405

[16] He Y Wang H Yu Z Yin J Jiang Y Zhou D. Unilateral biportal endoscopic versus uniportal full-endoscopic for lumbar degenerative disease: a meta-analysis. J Orthop Sci. (2024) 29(1):49–58. 10.1016/j.jos.2022.10.019 36437152

[17] Haibier A Yusufu A Hang L Abudurexiti T. Comparison of clinical outcomes and complications between endoscopic and minimally invasive transforaminal lumbar interbody fusion for lumbar degenerative diseases: a systematic review and meta-analysis. J Orthop Surg Res. (2024) 19(1):92. 10.1186/s13018-024-04549-7 38281015 PMC10821211

[18] Ito F Ito Z Shibayama M Nakamura S Yamada M Yoshimatu H. Step-by-step sublaminar approach with a newly-designed spinal endoscope for unilateral-approach bilateral decompression in spinal stenosis. Neurospine. (2019) 16(1):41–51. 10.14245/ns.1836320.160 30943706 PMC6449824

[19] Huang X Wang W Chen G Guan X Zhou Y Tang Y. Comparison of surgical invasiveness, hidden blood loss, and clinical outcome between unilateral biportal endoscopic and minimally invasive transforaminal lumbar interbody fusion for lumbar degenerative disease: a retrospective cohort study. Bmc Musculoskel Dis. (2023) 24(1):274. 10.1186/s12891-023-06374-1 PMC10088165 37038129

[20] Maaya A Hwa Eum J. Learning curve complications and management strategies in unilateral biportal endoscopic spine surgery: a case series. Cureus J Med Science. (2025) 17(12):e98479. 10.7759/cureus.98479 PMC12678202 41357726

[21] Santander XA Stienen MN Motov S Quintanilla HU Pérez EG. Complications and their prevention in unilateral biportal endoscopy: a systematic review with narrative insights and practical management algorithms. Acta Neurochir. (2026) 168(1):27. 10.1007/s00701-026-06784-9 41629728 PMC12868076

[22] Lee HG Kang MS Kim SY Cho KC Na YC Cho JM. Dural injury in unilateral biportal endoscopic spinal surgery. Glob Spine J. (2021) 11(6):845–51. 10.1177/2192568220941446 PMC8258823 32762357

[23] Sato K Konno S Yabuki S Mao GP Olmarker K Kikuchi S. A model for acute, chronic, and delayed graded compression of the dog cauda equina. Neurophysiologic and histologic changes induced by acute, graded compression. Spine. (1995) 20(22):2386–91. 10.1097/00007632-199511001-00003 8578388

[24] Kawakami M Takeshita K Inoue G Sekiguchi M Fujiwara Y Hoshino M. Japanese Orthopaedic Association (JOA) clinical practice guidelines on the management of lumbar spinal stenosis, 2021 - secondary publication. J Orthop Sci. (2023) 28(1):46–91. 10.1016/j.jos.2022.03.013 35597732

[25] Staszkiewicz R Gładysz D Gralewski M Bryś K Garczarek M Gadzieliński M. Usefulness of detecting brain-derived neurotrophic factor in intervertebral disc degeneration of the lumbosacral spine. Med Sci Monitor. (2023) 29:e938663. 10.12659/MSM.938663 PMC9854178 36642939

[26] Pao J. Preliminary clinical and radiological outcomes of the “no-punch” decompression techniques for unilateral biportal endoscopic spine surgery. Neurospine. (2024) 21(2):732–41. 10.14245/ns.2448376.188 38955542 PMC11224751

[27] Kim J Choi D Park EJ. Clinical and radiological outcomes of foraminal decompression using unilateral biportal endoscopic spine surgery for lumbar foraminal stenosis. Clin Orthop Surg. (2018) 10(4):439–47. 10.4055/cios.2018.10.4.439 30505412 PMC6250968

[28] Sun W Wang J Hu Y Tao J Yang C. Efficacy and safety of unilateral biportal endoscopy compared with transforaminal route percutaneous endoscopic lumbar decompression in the treatment of lumbar spinal stenosis: minimum 1-year follow-up. J Pain Res. (2025) 18:1071–80. 10.2147/JPR.S493602 40061433 PMC11890016

[29] Patel K Harikar MM Venkataram T Chavda V Montemurro N Assefi M. Is minimally invasive spinal surgery superior to endoscopic spine surgery in postoperative radiologic outcomes of lumbar spine degenerative disease? A systematic review. J Neurol Surg Part A. (2024) 85(2):182–91. 10.1055/a-2029-2694 36746397

[30] Zhibin L Zhantian A. Comparison of the efficacy between unilateral biportal endoscopic transforaminal lumbar interbody fusion (UBE-TLIF) and microendoscopic discectomy (MED)-assisted minimally invasive transforaminal lumbar interbody fusion (MIS-TLIF) in the treatment of mild single-level lumbar spondylolisthesis. BMC Surg. (2025) 26(1):11. 10.1186/s12893-025-03391-w 41340112 PMC12781491

